# Strategies for improving home blood pressure monitoring among pregnant women with low adherence in Ghana

**DOI:** 10.1002/pmf2.70294

**Published:** 2026-04-13

**Authors:** Akosua Y. Oppong, Roxanne F. Harfmann, Betty A. Nartey, Clara Buxton, Cheryl A. Moyer, Jody Lori, Samuel A. Oppong, Emma R. Lawrence

**Affiliations:** ^1^ Department of Obstetrics and Gynecology Beth Israel Deaconess Medical Center Boston Massachusetts USA; ^2^ Department of Obstetrics and Gynecology University of Michigan Ann Arbor Michigan USA; ^3^ Department of Obstetrics and Gynecology Korle Bu Teaching Hospital Accra Ghana; ^4^ Department of Learning Health Sciences University of Michigan Ann Arbor Michigan USA; ^5^ University of Michigan School of Nursing Ann Arbor Michigan USA; ^6^ Department of Obstetrics and Gynaecology University of Ghana Medical School Accra Ghana

**Keywords:** adherence, digital health, Ghana, home blood pressure monitoring, hypertensive disorders of pregnancy, maternal health, qualitative research

## Abstract

**Introduction:**

Hypertensive disorders of pregnancy (HDP) remain a leading cause of maternal morbidity and mortality globally, with a disproportionate burden in low‐ and middle‐income countries (LMICs) such as Ghana. In high‐income settings, home blood pressure (BP) monitoring has emerged as an effective tool for early detection of HDP; however, limited data exist regarding its feasibility and acceptability in LMICs. This qualitative study sought to explore the experiences of women in Ghana who demonstrated low adherence to a home BP monitoring intervention during pregnancy.

**Methods:**

This study was conducted at Korle Bu Teaching Hospital (KBTH), a tertiary care hospital in Accra, Ghana. Participants were 16 postpartum women, purposively sampled from an overarching cohort study of home BP monitoring based on low adherence, defined as recording BP values ≤ 4 days per week on average. Semi‐structured interviews were conducted in English or Twi. Interview guide questions focused on participants’ experiences recording their BPs, specifically eliciting challenges and perceived benefits. Interviews were audio‐recorded, translated, transcribed, coded in Dedoose using an iteratively developed codebook, and thematically analyzed.

**Results:**

Participants had a mean age 30.4 years, 87% were married, and 69% had previously given birth. There were 29 codes grouped into four comprehensive themes: (1) disconnect between understanding and action, (2) barriers to adherence, (3) facilitators, and (4) suggestions for improvement. While many women understood the importance of BP monitoring in pregnancy and acknowledged its benefits, for some, there was dissonance between their understanding and behavior. Barriers included competing health priorities and familial responsibilities. Women emphasized familial support as a key facilitator to monitoring their BPs and suggested inclusion of their spouses and family members in further training. Finally, several women noted that frequent reminders, along with a machine with automatic data capturing, would facilitate adherence to at‐home monitoring.

**Conclusion:**

Though participants recognized that home blood pressure monitoring was important, several barriers, including familial responsibilities and physical health, prevented them from adhering to daily checks. Incorporating culturally relevant digital reminders, simplified logging methods, and support from family may strengthen adherence. Future work will adapt and evaluate a smartphone‐based reminder and data‐capture platform informed by stakeholder input.

## 1 INTRODUCTION

Hypertensive disorders of pregnancy (HDP), including gestational hypertension, pre‐eclampsia, and eclampsia, affect approximately 10% of pregnancies worldwide and remain a leading cause of maternal mortality in low‐ and middle‐income countries (LMICs). Complications of HDPs include stroke, preterm birth, placental abruption, renal failure, and death [1]. In Ghana, recent studies have reported that approximately 21%–37% of pregnancies are complicated by HDP [2, 3], and an estimated 25% of maternal deaths are attributable to these disorders [4].

Comparatively, in the United States, the prevalence of HDPs was 15.9% in 2019 [5], contributing to approximately 8% of maternal deaths [6]. In high‐income countries, early detection and diagnosis of HDP are central to antenatal care, allowing for timely intervention and closer monitoring of disease progression. Home blood pressure (BP) monitoring, in particular, is widely implemented among pregnant individuals at elevated risk for HDP or worsening chronic hypertension between clinic visits. Successful home BP monitoring relies on consistent adherence, accurate recognition of elevated readings, and prompt communication with healthcare providers for management [7, 8].

Home BP monitoring is widely used in high‐income countries in various populations, including pregnant individuals [9, 10]. However, there are limited data on its utility in LMICs [10, 11]. Previous studies in Ghana have demonstrated that pregnant women generally express interest in home BP monitoring due to a desire for greater control and awareness of their health. However, barriers to frequent and consistent home BP monitoring, such as time constraints, psychosocial stress, and competing responsibilities, were reported [12]. These challenges are likely compounded in LMICs like Ghana, where limited health literacy, socioeconomic constraints, and variable engagement in antenatal care may further impede adherence with home BP monitoring [13].

The subgroup of women who struggle most with completing frequent home BP monitoring may have unique challenges or circumstances that drive their low adherence. Further, identifying these barriers may provide actionable targets to support these least successful women to improve consistent engagement in home monitoring. Therefore, additional research is needed to understand the contextual factors influencing adherence to home BP monitoring among pregnant women in Ghana who struggled most with home BP monitoring. The present study aims to explore the experiences of women with low adherence to at‐home BP monitoring, identify barriers and facilitators to consistent monitoring, and assess recommendations to enhance implementation.

## 2 METHODS

### 2.1 Setting

This study was conducted at the Korle Bu Teaching Hospital (KBTH), a tertiary care hospital situated in Accra, the capital city of Ghana. KBTH provides antenatal care, labor and delivery, and postpartum care for a wide range of pregnant women, including those with low‐risk and high‐risk pregnancies. Hypertensive disorders of pregnancy, including pre‐eclampsia and eclampsia, constitute a large proportion of the cases of maternal morbidity and mortality at KBTH [14, 15].

### 2.2 Participants

The study included 16 postpartum women who had participated in a home BP monitoring intervention throughout their pregnancy and exhibited low adherence to the intervention. Inclusion criteria were completion of an overarching cohort study on home BP monitoring, age of at least 18 years, fluency in English, Twi, or Ga, having attended antenatal care and delivery at KBTH, and low adherence with home BP monitoring. Low adherence was defined as measuring and recording their BP on average less than or equal to 4 out of the 7 days in a week.

### 2.3 Recruitment

**FIGURE 1:**
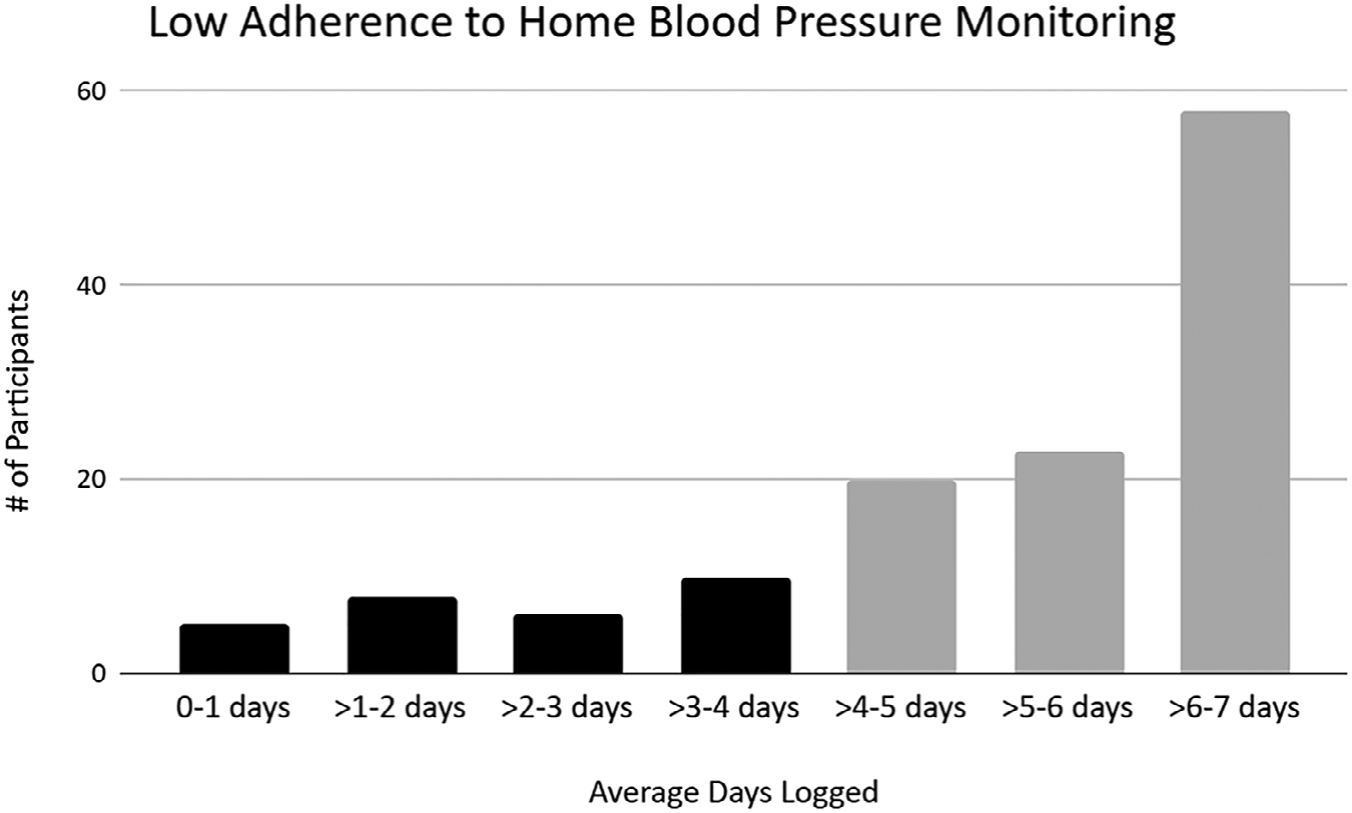
Participants with low adherence to home blood pressure monitoring.

Among 132 total participants engaged in the overarching home BP monitoring intervention, 29 participants were found to have low adherence, defined here as four or fewer days logged per week on average (Figure 1). Seven of the individuals were unable to be contacted via telephone, and one individual had been interviewed prior for a different arm of the study. Of the remaining 21, 19 participants agreed to be interviewed, and 16 interviews were conducted. Recruitment and interviews continued until thematic saturation was achieved. Saturation was determined by the researchers, who conferred and agreed that there were no new emerging patterns or themes from the interviews.

### 2.4 Approach

This qualitative study was conducted among a key group of participants with low adherence who had completed an overarching cohort study of home BP monitoring. The cohort study involved the recruitment of pregnant participants at their initial antenatal visit at KBTH. Enrolled participants underwent in‐person training on the use of an automatic microlife BP monitor, validated in pregnancy [16]. The training included videos, script‐based teaching, and hands‐on practice, and covered how to correctly measure BP and reviewed normal versus high BP values. Participants were instructed to check their BP at home once a day until delivery, record the BP in a written log, and call a study midwife for advice if any BP measurements were above 140/90. The written logs given to participants contained pictorial symbols reminding participants how to correctly check their BP and to distinguish between high versus normal values. Logs were collected from participants when they presented for their routine antenatal visits. All logs were reviewed, and data on frequency and timing of BP logging were extracted. Participants with low adherence, defined as those who recorded BP values less than or equal to 4 out of the 7 days of a week, on average, cumulatively over their recording window, were identified.

The participants with low adherence were recruited for interviews that occurred in December 2024 and January 2025. Interviews were semi‐structured and conducted in person either at a private research room at KBTH or a convenient location of the participants' choice. Interviews were one‐on‐one. The interviewer was a Ghanaian‐American Obstetrics and Gynecology resident who had undergone training in qualitative methods through a Master of Public Health degree and review with the primary researcher. Participants were given the choice of performing the interviews in either English, Twi, or Ga, the three main languages spoken in Accra; 13 interviews were conducted in English, three were conducted in Twi, and none in Ga. An interview guide was utilized to conduct the interviews and was structured to start with open‐ended questions and follow up with more specific prompts. Questions focused on patients' experiences with measuring their BPs at home, challenges they faced in remaining consistent, perceived benefits of checking their BPs at home, and suggestions for improving adherence in future iterations of the project. Content for the interview guide was based on the experience of the research team with home BP monitoring and findings from their prior research [12, 17]. Interviews lasted approximately 30 min and were audio‐recorded in full. No additional field notes were taken.

### 2.5 Analysis

Interviews recorded in Twi were translated into English and transcribed using Microsoft Word. Interviews recorded in English were transcribed using a transcription service, GoTranscript. Transcripts were reviewed by two researchers, and a codebook was developed using an iterative approach. First, transcripts were separately reviewed by researchers who each identified key ideas. Then, transcripts were compared to develop common keyword phrases, and a preliminary codebook was developed. The preliminary codebook was tested against new sets of transcripts until it stabilized to ensure that the coding was similar between researchers and no changes in codes were needed. The transcripts were then uploaded to Dedoose and coded using the final codebook. Quotes were then downloaded by code, and thematic analysis was conducted following the Attride‐Stirling approach to identify basic, organizing, and global themes [18]. Sociodemographics were descriptively summarized.

## 3 RESULTS

Participant demographics are described in Table 1. Interviews were initially coded into 29 codes grouped into six thematic categories. Codes were later grouped into four comprehensive themes: disconnect between understanding and action, barriers, facilitators, and suggestions to improve adherence.

**TABLE 1:** Sociodemographics of participants.

Participant demographics	% (*n*)
Age (years, m [range])	30.4 (22–39)
Highest level of education completed	
None	0 (0)
Primary school	6.3 (1)
Junior high school	6.3 (1)
Senior high school	31.3 (5)
Tertiary	56.3 (9)
Monthly household income in Cedis	
<650 (<$59)	31.3 (5)
650–1000 ($59–91)	6.3 (1)
1000–5000 ($91–457)	56.3 (9)
>10,000 (>$913)	6.3 (1)
Insurance	
Public	93.7 (15)
Private	6.3 (1)
Marital status	
Married	87.5 (14)
Not married	12.5% (2)
Number of births	
0	31.3 (5)
1	43.8 (7)
2	18.8 (3)
3	6.3 (1)
Prior HDP	6.3 (1)
Chronic hypertension	0 (0)

### 3.1 Disconnect between cognitive value of home BP monitoring and taking action

Overall, women found home BP monitoring to be beneficial. They recognized the importance of awareness surrounding BP readings and the utility of regular monitoring during pregnancy.

…it's important because for you to live without checking your bp is bad, you have to be checking your bp, for a pregnant woman you have to be checking your bp for you to know where you are. (Age 27, primary school education)

One woman mentioned that measuring her BP allowed her to know whether or not she needed medical attention:

Because you know your status, you know where you are standing, whether you are high, you are low, you know it is. If you are high, it means you have to be attended to, then you have to go to the hospital. (Age 23, senior high school education)

Women were aware of the poor outcomes associated with high BP and thus acknowledged the importance of regular assessments.

It's a good thing to do. Eventually, it helps because maternal BP check during pregnancies is important to diagnose preeclampsia and all those things. (Age 34, tertiary education)

I've heard that BP kills, so it's good to check your BP from time to time so that you'll be aware of how your BP is, whether it's gone up or it's down. (Age 23, senior high school education)

Though women had low adherence to monitoring their BP in their current pregnancy, all women unanimously expressed desire to monitor in subsequent pregnancies.

…For me, if I'm getting pregnant again, I would love to do it again. (Age 23, senior high school education)

However, while many women in this sample demonstrated understanding of the importance of BP monitoring in pregnancy and overwhelmingly acknowledged the benefit of home BP monitoring during pregnancy, some women demonstrated a dissonance between their understanding and actual behavior. For example, when asked about things that make home BP monitoring difficult, one woman said:

If they know that checking your BP is like… it's killing most of the people out there, pregnant women…If they know that there's danger in being low or high, it will give them more attention to the whole situation. (Age 31, tertiary education)

Yet, despite understanding the dangers of abnormal BP during pregnancy, when this same woman was asked why she stopped monitoring her BP during pregnancy, she responded:

Do I even know? I just thought I was okay because every time I was checking, it was okay. (Age 31, tertiary education)

### 3.2 Barriers to home blood pressure monitoring adherence

Participants identified many factors that prevented them from regularly monitoring their BP at home. When compared to our prior work identifying barriers and facilitators among pregnant women with all levels of adherence and receiving antenatal care at KBTH (published in Newman et al.^12^), many of the barriers identified by women in the current low adherence sample were consistent with the larger group (Table 2).

**TABLE 2:** Comparison of barriers between patients with all levels of adherence (Newman et al.^12^) and patients with low adherence (current study).

Barrier identified in past work among women with all levels of adherence (Newman et al.[12])	Corresponding barrier reported by women with low adherence in the current study	Example quote from current study
Time, stress, and daily responsibilities	Time and daily activities	“Sometimes my children will be worrying me at home, and I don't have time for the machine.” (Age 27, primary school education)
Perceived importance of BP in pregnancy	Perceived unimportant	“If you don't know the importance of what you are doing, you will not attach this seriousness to it.” (Age 34, tertiary education)
Role of family	Lack of support	“Sometimes getting up to go and sit in the chair is difficult because I live alone and I don't get anyone to tie the belt for me. Mostly I have to do it myself.” (Age 31, tertiary education)
Capability of performing BP monitoring	Lack of knowledge	“There are some people who don't have understanding of what they are doing so I think that is the main reason why a lot of them may not be monitoring their BP.” (Age 31, senior high school education)
Convenience of monitoring process	Machine access issues	"I traveled. I forgot the machine. I forgot about it. That was what caused the break." (Age 28, tertiary education)

However, among low adherence women, there appear to be unique factors that may have specifically impeded their adherence. Some women noted how their current physical health prevented them from monitoring their BP at home.

At some point, it was when the fibroid pain became very intense. Then it was taking over my entire morning. The little time I have, I want to rest. The last thing you think about is checking your BP because you just want the pain to reduce a little bit so you can catch some sleep. (Age 31, tertiary education)

For other women, their current mental state, especially feeling unmotivated, also acted as a barrier to home BP monitoring.

For me, that was the challenges I was having…Laziness is number one. I think that's it. (Age 23, senior high school education)

Several women related their low adherence to repeated normal BP readings, even though, as previously stated, most women recognized the importance of BP monitoring in pregnancy. Frequent normal BP readings for these women were demotivating to continue monitoring:

Because when I checked, it always looked normal to me. I was within the normal value, so sometimes I say, ‘Oh, I check yesterday, it was fine, so relax’. (Age 39, tertiary education)

Getting the normal value really got me happy and so I wasn't doing it consistently because I knew there's nothing alarming about my BP. I'm getting a good BP. (Age 35, tertiary education)

### 3.3 Facilitators to home blood pressure monitoring adherence

In addition to barriers, some pregnant women talked about factors that helped them to monitor their BP at home. Again, when comparing to our prior work (published in Newman et al.^12^), many of the facilitating factors reported by women in the current low adherence subgroup aligned with facilitators that have been previously identified by pregnant women with all levels of adherence (Table 3).

**TABLE 3:** Comparison of facilitators between patients with all levels of adherence (Newman et al.[12]) and patients with low adherence (the current study).

Facilitator identified in past work among women with all levels of adherence (Newman et al.[12])	Corresponding facilitator reported by women with low adherence in the current study	Example quote from current study
Perceived importance of BP in pregnancy	Desire to be informed	“I think it's good to be checking the BP because it helps you stay alert and to know whether you're not in danger or you're okay.” (Age 23, senior high school education)
Role of family	Family support	“Sometimes, I forget then my husband will alert me by asking me if I have checked then I go and check.” (Age 32, junior high school education)
Capability of performing BP monitoring	Training and knowledge	“[Training] was insightful because it will teach you how to use a monitor, I was shown a video. I was shown a video, and it's helped me to know what to do.” (Age 34, tertiary education)
Convenience of monitoring process	Access to monitor	“Monitoring at home, you have access to the machine, at any point in time you can monitor it, but when you don't have it at home, maybe you don't feel okay and perhaps you think it might be your BP. You have to maybe walk to the nearest pharmacy or hospital to do it. You might not have the energy or the strength to do so, but when you have it at home with you, you do it.” (Age 38, tertiary education)

Pregnant women with low adherence in the current sample also identified reminders as a facilitating factor to home BP monitoring. While past work notes the role of family and their ability to remind pregnant women to monitor their BP daily,^12^) women in this sample also explicitly reported that visit and call with midwives helped to remind them to monitor their BP at home:

The [midwife] calling is very good. Sometimes you might forget, and then it just reminds you that you have to do this thing, not only for the research purposes, but for your own good. (Age 28, tertiary education)

### 3.4 Suggestions to improve adherence

When asked about ways to improve adherence, the most common suggestions related to reminders, improvements in technology, and family involvement.

For those who recognized the importance of checking their BP, they noted that regular reminders, through more frequent calling by the midwives, may have motivated them to regularly check their BP.

…They sometimes call and check on me, how I'm faring, and they even said if there's any problem, you can call on them. I think there could be more. That's more of maybe they call and maybe in a week, three or four times, maybe they prompt you, you remember. (Age 38, tertiary education)

Others note that group messages utilizing a platform such as WhatsApp would have increased their likelihood to monitor their BP.

The text will be okay. If we can have the WhatsApp group, every morning, try and check your BP. If you have that WhatsApp,… it will come you know that this is coming for BP. (Age 32, senior high school education)

Beyond reminders to check their BP, some women found physically recording their BPs in the paper log to be difficult. Women suggested that reducing this burden could help adherence, with some promoting integration of their phones or recommending a more advanced machine that automatically records and retains the BP reading.

You can try to find a means of doing it with our phones because somebody can misplace their hard copy. With a phone, [it] can be easier. (Age 34, tertiary education)

If there are more advanced machines, like when you check, it's recorded by itself. You don't have to manually record. That could have made it easier. (Age 26, tertiary education)

In addition to reminders and improved technology, women emphasized the importance of family involvement in their adherence to monitoring their BPs. They recognized that more active involvement of their spouses and extended family members could encourage them to check their BP more regularly.

As I said earlier, if I should get pregnant again and I am given a machine, I will get someone to stay with me who will help me when I am weak and not able to check myself. (Age 31, senior high school education)

…If you are doing something and you get your partner involved, it makes you know that you are not alone in the situation. At least you are motivated to do it. (Age 35, tertiary education)

Though more than half the women had completed some level of tertiary education, many did not seem to correlate the risk of hypertension with its possible manifestation in their own pregnancy.

Thus, some stressed the importance of additional education regarding gestational hypertension with a specific emphasis on the risk of health complications.

From your end to the person, I think more of education. Other than that, you can't go home and do some of the activities, the chores for her. It should be more of education. The education should sink more. Then to let her know on the dangers of BP so that at least he or she will be frightened a bit to pave way for it. (Age 35, tertiary education)

Finally, although BP monitoring was ultimately to the benefit of the individuals, some mentioned incentivization as a method to improve adherence.

Increase the money, [chuckles] or maybe you can say that if you do a minimum of 10 readings, you get this amount. If you do a minimum of 20 readings, you get this amount, so you can increase the amount, make it more exciting, and to make it attractive. If I'm doing a hundred readings and I come and I'm getting 20, and I do one and I get 20 of these, I wouldn't really spend time. I think that would be a good thing. (Age 34, tertiary education)

## 4 DISCUSSION

Our findings identify barriers and facilitators to home BP monitoring among pregnant women in Ghana with low adherence to daily monitoring. Several barriers reported by women in the current study were consistent with past work [12]; however, women also identified barriers that may be specific in the context of low adherence to home BP monitoring. Specifically, although women recognized the importance of monitoring in pregnancy and understood the risks associated with high BP in pregnancy, there was significant discord between knowledge and action, as many of them were non‐adherent to home BP monitoring. This disconnect often stemmed from reassurance by normal BP readings, which seemed to lead to decreased motivation to continue daily checks. Other women cited physical discomfort and fatigue as reasons for non‐adherence. These findings emphasize that knowledge alone is insufficient to sustain self‐monitoring and may highlight the possible need to address behavioral components alongside education [19]. Further education surrounding the dynamic nature of HDP, particularly its common onset in the late third trimester or postpartum period, may be vital in encouraging pregnant women to regularly monitor their BPs [20]. Educational reinforcement could be implemented in antenatal counseling or in training sessions for home BP cuff usage.

In addition to barriers, women identified several facilitators that supported adherence, such as perceived health benefits, family support, and convenience of access to personal BP cuffs, which aligned with facilitators reported in previous work [12]. A novel facilitator that emerged in this low‐adherence population was the motivational role of weekly check‐in calls from midwives involved in the study. For them, these calls served as a reminder and accountability. This reflects the importance of communication in sustaining adherence in self‐monitoring programs, particularly in settings with low health literacy and structural barriers limiting engagement.

Facilitating factors that made home BP monitoring easier for pregnant women highlight opportunities to improve the present intervention, particularly for pregnant women who demonstrated low adherence to daily monitoring. Women suggested reminders to complete daily monitoring or integration of technology to alleviate the logging burden would help improve adherence to the intervention. Regular reminders and digital monitoring platforms have been incorporated into other self‐monitoring BP interventions for pregnant women at risk for HDP. In one study, pregnant participants were asked to report their BP for three consecutive weeks (Monday through Friday) and standard daily alerts (i.e., push notifications) were sent to remind participants to take their BP measurements [21, 22]. Findings from this study indicated that pregnant women utilizing the digital health platform to monitor their BP at home had fewer antenatal hypertension‐related admissions [21].

In a retrospective study, pregnant women at risk for HDP completed remote monitoring via a wireless BP monitor and were asked to make two BP measurements daily until hospital admission or delivery [23]. Compared to women in a usual care group, pregnant women monitoring their BP at home had fewer prenatal ward admissions and hospitalizations up to delivery [23]. The findings suggest that home BP monitoring may help identify abnormal BP measurements or symptoms in the home environment, possibly earlier in pregnancy due to consistent monitoring, and thus prevent worsening of hypertensive disease. Home monitoring's ability to improve blood pressure control, reduce target organ damage, and decrease cardiovascular risk has been shown in its application in individuals with chronic hypertension [24, 25].

While the technology may not yet be widely available in Ghana, adapting low‐cost, context‐appropriate digital reminder systems could strengthen implementation. Application‐based interventions should be designed with stakeholder input, ensuring inclusion of perspectives from pregnant women, midwives, and healthcare providers to ensure usability and integration within the existing antenatal care systems. Future areas for research include adapting and assessing the feasibility of a smartphone application for home BP monitoring using input from important stakeholders. This approach could address many of the barriers identified in this study, including forgetfulness and manual burden. The potential benefits of automated data logging on improved adherence need to be weighed against the cost of technology [26]. Further, as women highlighted social support as a facilitator, formalizing family involvement in training and monitoring could potentially improve adherence.

Strengths of our study include the qualitative design that allowed for valuable insights into the behavioral and contextual factors influencing adherence and highlighting areas for improved intervention design. Though it is important to recognize that these behaviors are among a particular group of women who were specifically selected based on their real‐world experience with non‐adherence to measuring their BPs at home. Limitations of our study include all participants being recruited from a tertiary care facility. Therefore, their experiences may not fully reflect those of women in rural or low‐resource settings.

## 5 CONCLUSION

In summary, the study provides insight into the behavioral and contextual aspects of adherence to home BP monitoring among pregnant women in Ghana. Despite recognizing the importance of BP monitoring in pregnancy, the women note many barriers to adherence, including motivation. The data highlight that adherence is shaped by knowledge and access as well as motivation, convenience, and social support. Strengthened educational messaging, structured technological reminders, and integration of family and community support could enhance the sustainability of home BP monitoring interventions. Integrating culturally relevant tools can be instrumental in overcoming barriers.

## CONFLICT OF INTEREST STATEMENT

The authors declare no conflicts of interest.

## ETHICS STATEMENT

Ethical approval was granted from KBTH (KBTH‐IRB 00098/2021) and the University of Michigan (HUM00200589). An incentive of local currency cash valued at approximately $10 was provided for participation. Written informed consent was obtained from all participants.

## Data Availability

The data that support the findings of this study are available from the corresponding author upon reasonable request.
